# Subclinical Auditory Neural Deficits in Patients With Type 1 Diabetes Mellitus

**DOI:** 10.1097/AUD.0000000000000781

**Published:** 2019-04-27

**Authors:** Arwa AlJasser, Kai Uus, Garreth Prendergast, Christopher J. Plack

**Affiliations:** 1 Department of Rehabilitation Sciences, College of Applied Medical Sciences, King Saud University, Riyadh, Saudi Arabia; 2 School of Health Sciences, Manchester Centre for Audiology and Deafness, University of Manchester, Manchester, United Kingdom; 3 Department of Psychology, Lancaster University, Lancaster, United Kingdom.

**Keywords:** Frequency-following response, Speech in noise, Subclinical hearing loss, Temporal coding, Type 1 diabetes

## Abstract

**Objectives:**

Diabetes mellitus (DM) is associated with a variety of sensory complications. Very little attention has been given to auditory neuropathic complications in DM. The aim of this study was to determine whether type 1 DM (T1DM) affects neural coding of the rapid temporal fluctuations of sounds, and how any deficits may impact on behavioral performance.

**Design:**

Participants were 30 young normal-hearing T1DM patients, and 30 age-, sex-, and audiogram-matched healthy controls. Measurements included electrophysiological measures of auditory nerve and brainstem function using the click-evoked auditory brainstem response, and of brainstem neural temporal coding using the sustained frequency-following response (FFR); behavioral tests of temporal coding (interaural phase difference discrimination and the frequency difference limen); tests of speech perception in noise; and self-report measures of auditory disability using the Speech, Spatial and Qualities of Hearing Scale.

**Results:**

There were no significant differences between T1DM patients and controls in the auditory brainstem response. However, the T1DM group showed significantly reduced FFRs to both temporal envelope and temporal fine structure. The T1DM group also showed significantly higher interaural phase difference and frequency difference limen thresholds, worse speech-in-noise performance, as well as lower overall Speech, Spatial and Qualities scores than the control group.

**Conclusions:**

These findings suggest that T1DM is associated with degraded neural temporal coding in the brainstem in the absence of an elevation in audiometric threshold, and that the FFR may provide an early indicator of neural damage in T1DM, before any abnormalities can be identified using standard clinical tests. However, the relation between the neural deficits and the behavioral deficits is uncertain.

## Abstract 2

Supplemental Digital Content is available in the text.

## INTRODUCTION

Diabetes mellitus (DM) is a metabolic disorder characterized by hyperglycemia, with disturbances in the metabolism of carbohydrates, fat, and protein resulting from defects in insulin secretion, insulin action, or both. Several pathogenic processes may result in the development of DM. These include autoimmune destruction of beta cells in the pancreas, resulting in insulin deficiency, as seen in type 1 DM (T1DM), as well as other factors that result in resistance to the action of insulin on the target tissues, which is the case in the majority of type 2 DM (T2DM) patients (Alberti & Zimmet 1998).

The investigation of the relation between DM and disorders of the auditory and vestibular systems has been going on for over a century (Jordão 1857, cited in McQueen et al. 1999); however, the association remains controversial and conflicting results are reported in the literature. The results of some animal and human studies point to changes in anatomical structures such as increased thickness of inner ear and basilar membrane vessels (Costa 1967; Smith et al. 1995; Fukushima et al. 2006; Kariya et al. 2010), loss of outer hair cells (Nakae & Tachibana 1986; Triana et al. 1991; Raynor et al. 1995; Fukushima et al. 2006), and demyelination of the auditory nerve (AN) (Makishima & Tanaka 1971). Diabetic abnormalities have also been demonstrated in the central auditory pathways; however, the pathogenesis is still unclear (Reske-Nielsen et al. 1966; Luse et al. 1970; Makishima & Tanaka 1971; Jakobsen et al. 1987; Dejgaard et al. 1991).

Studies of the hearing health of DM patients have tended to focus on pure-tone audiometry (PTA). Meta-analyses have found that the presence of DM roughly doubles the odds of developing an audiometric hearing loss, with a greater effect at high frequencies (Horikawa et al. 2013; Akinpelu et al. 2014a). However, audiometric hearing loss is not an inevitable consequence of DM. Some studies report no hearing loss compared with sex- and age-matched controls (Friedman et al. 1975; Dalton et al. 1998).

Although neuropathy is one of the more common complications in DM, affecting up to 50% of patients (Boulton et al. 2004), little attention has been given to neuropathic complications in DM involving the AN and central auditory pathways. These deficits, even in the absence of an elevation in audiometric threshold, may result in listening difficulties (Moore 2008). Studies using the auditory brainstem response (ABR) have found some differences between the ABR waveforms of DM patients and those of sex- and age-matched controls (Parving et al. 1990; Bayazit et al. 2000; Lisowska et al. 2001; Frisina et al. 2006; Konrad-Martin et al. 2010). The amplitude of wave I of the ABR, which reflects AN function, is often little affected in normal-hearing DM patients compared with controls (Al-Azzawi & Mirza 2004; Spankovich et al. 2017). Although there are reports of increased wave I latency in DM patients, even in the presence of normal audiometric hearing (Al-Azzawi & Mirza 2004; Durmus et al. 2004; Acar et al. 2012), a recent meta-analysis found no significant effect (Akinpelu et al. 2014a). The evidence for central auditory neural dysfunction is stronger. Increases in central wave latencies and increased I to V, III to V, and I to III interpeak intervals (Martini et al. 1987; Parving et al. 1990; Durmus et al. 2004; Vaughan et al. 2007; Konrad-Martin et al. 2010; Rance et al. 2014, 2016), as well as reduced amplitudes for waves III and V (Rance et al. 2014), have been reported. These results are considered a sign of delayed conduction of neural response or loss of neural synchrony and suggest that DM is associated with an increase in neural transmission time, possibly as a result of demyelination.

Very few studies have investigated the behavioral consequences of neuropathic complications in DM patients. These studies have identified trends of subclinical temporal processing difficulties, leading to perceptual difficulties in challenging acoustic environments (Frisina et al. 2006; Rance et al. 2014, 2016; Silva et al. 2017). Some studies have found that speech discrimination scores in quiet and in noise were lower in DM patients with normal PTA thresholds compared with controls, with a greater difference in the speech-in-noise conditions (Kakarlapudi et al. 2003; Rance et al. 2014; Silva et al. 2017).

A review of the literature shows little agreement about the impact of DM on auditory function, let alone specifically on the involvement of the AN and central neural pathways, and reveals the need for further research, using more sensitive assessment methods with the ability to detect significant subclinical changes in the auditory system. The overall aim of the present study was to determine whether T1DM affects processing in the AN and brainstem, in particular coding of the temporal aspects of sounds, and how any deficits may impact on behavioral performance.

The main limitation shared by most of the published studies that have investigated the relation between DM and hearing deficits is the choice of participant samples, exemplified by lack or inadequacy of matched control groups, mixing of T1DM and T2DM patients, and use of elderly DM participants. Unmeasured or imprecisely assessed potential confounding factors, such as participants’ age, type of DM, presence or absence of DM complications, and comorbidity, may have caused a multitude of conflicting outcomes and made it difficult to determine the possible associations between these variables, and consequently the physiological basis of the auditory dysfunction in DM. In an attempt to avoid such confounds, strict recruitment criteria were used in the present study to only include young (aged 18 to 35 years) T1DM patients with binaurally hearing thresholds of 20 dB HL or better for frequencies ranging from 500 to 4000 Hz. The study also used tight pair matching to controls with respect to age, sex, and audiometric thresholds. Moreover, DM-related factors such as DM duration and the presence of clinically diagnosed neuropathy and retinopathy were obtained with a secondary aim of investigating their effects on the results of the experimental measures used in the study. It was hypothesized that patients with diabetic neuropathy or retinopathy are more likely to present with neuropathic complications involving the AN and central auditory pathways.

In addition to the ABR, the test battery included the electrophysiological frequency-following response (FFR). The FFR reflects sustained neural activity, phase locked to the cycles of the stimulus waveform. Two types of information are represented: the envelope, which corresponds to slow variations in overall amplitude over time, and the temporal fine structure (TFS) which corresponds to the rapid individual variations in sound pressure (Moushegian et al. 1973; Moore 2008). Accurate encoding of both the envelope and TFS of a stimulus is believed to be important for understanding speech, especially in noisy environments (Sachs et al. 1983; Rosen 1992; Lorenzi et al. 2008). The FFR is thought to originate mainly from brainstem generators, although there may also be AN and cortical contributions (Bidelman 2015; Coffey et al. 2016). To the authors’ knowledge, no study has explored DM-related auditory deficits with the use of the FFR, although the FFR has been shown to be sensitive to pathological changes in the AN in other patient populations (McAnally & Stein 1996; Russo et al. 2009; Basu et al. 2010; Jafari et al. 2015).

The test battery also included speech-in-noise tests, and behavioral tests assumed to be dependent on temporal coding: interaural phase difference (IPD) discrimination, and the frequency difference limen (FDL). The interaural timing difference, which for periodic and ongoing tones such as pure tones translates to IPD, is the difference in arrival time of a sound between the two ears. Interaural timing difference and IPD are the most important cues to sound localization for most natural sounds in the environment in which low-frequency components are present (Wightman & Kistler 1992). The FDL is another commonly used behavioral measure of temporal coding. There is still debate as to whether pure-tone frequency discrimination depends on temporal or place coding cues at high frequencies, although temporal cues are probably used to perform the task at the frequency of 590 Hz used here (Sek & Moore 1995). (For reviews of pitch perception theories, see Moore 2012 and Plack 2018.)

Although self-report auditory disability measures are commonly used in hearing research, few studies have assessed DM individuals’ subjective experience of hearing disability to determine whether the postulated effects of DM on auditory function manifest in realistic listening situations. Using the Abbreviated Profile of Hearing Aid Benefit hearing/communication disability questionnaire, Rance et al. (2016) found that 19 school-age children with T1DM reported significantly greater difficulties, particularly in noisy or reverberant environments such as classrooms and playgrounds, compared with age- and sex-matched controls. In the present study, self-reported ability to hear in different everyday situations was measured using the Speech, Spatial and Qualities (SSQ) hearing scale.

The primary research questions were as follows:

1. Do T1DM patients show evidence of cochlear neuropathy or central neural dysfunction?
2. Is T1DM associated with poorer performance on behavioral tasks in the absence of an elevation in audiometric threshold?
3. Is T1DM associated with self-report of auditory disability in the absence of an elevation in audiometric threshold?

## MATERIALS AND METHODS

### Participants

The sample size was calculated based on a related pilot study with an effect size, *d*, of 0.49. This power calculation (G* power calculator, v3.1) suggested a minimum sample size of 27 participants per group to provide a statistical power value of 0.8 for a one-tailed prediction and an alpha level of 0.05 to detect a difference between the two groups, based on a paired samples *t* test. To allow for drop-out or larger than expected measurement variability, 30 participants per group were recruited. It is worth noting that the sample size adopted in this study is larger than in the two similar studies which were published after the start of the present study by Rance et al. (2014, 2016) (n = 10 and 19 per group, respectively). As discussed earlier, these studies were able to detect significant group differences between T1DM and the matched controls in all of the measures used, including ABR, speech-in-noise, and self-report measures. Thus, the sample size used in this study was expected to be sufficient to detect differences in these same measures. Sixty young audiometrically normal adults participated (binaural hearing thresholds for all participants were <20 dB HL for frequencies ranging from 500 to 4000 Hz). Thirty were T1DM participants (mean age, 26.8 years; range, 19 to 35 years; 22 females) (see Table in Supplemental Digital Content 1, http://links.lww.com/EANDH/A561, for the details of the 30 T1DM participants). The T1DM participants were pair-matched to 30 controls in terms of age, sex, and PTA threshold. For T1DM participants, T1DM diagnosis was confirmed through their consultant physicians or general practitioner, whereas each control participant reported that he/she was DM free; however, no measurement of blood glucose was taken to confirm the absence of DM in the control group. All participants had English as their first language.

**Fig. 1.**
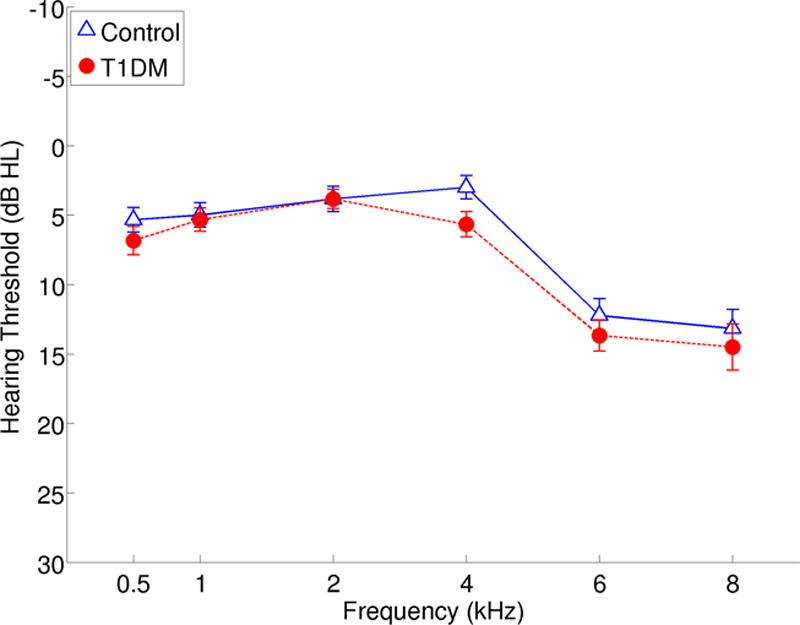
Mean air conduction audiometric thresholds of the test ears of the two groups. Error bars show SEs. T1DM indicates type 1 diabetes mellitus.

A decision was made at the beginning of the study to test the right ear of all participants, for monaural tests, unless the left ear average hearing threshold was at least 15 dB less than the right ear. The right ear was tested monaurally for all 60 participants. Criteria for matching T1DM and control participants were a difference in age of 11 months or less, and a difference in PTA thresholds of the test ear of 5 dB or less for each frequency at 0.5, 1, 2, and 4 kHz (see Table in Supplemental Digital Content 2, http://links.lww.com/EANDH/A562, for the details of the 30 matched pairs). However, it should be noted that although no efforts were made to match PTA thresholds at higher frequencies (6 and 8 kHz), no significant difference was found between the two groups in PTA thresholds of the test ears at 6 nor 8 kHz (N = 30, *z* = −1.20, *p* = 0.16 and *t* (29) = 0.97, *p* = 0.44, respectively; Fig. 1). The procedures were approved by the National Health Service (NHS) research ethics committee (reference number 12/NW/0319).

### Electrophysiological Measures

#### General Procedure •

All electrophysiological recordings were made in a single 2-hr session using TDT BioSig software. All stimuli were generated using MATLAB (MathWorks, 2010) and presented to the right ear via a TDT RP2.1 Enhanced Real Time Processor and HB7 Headphone Driver with the participant’s left ear plugged using a foam plug. Recordings were made with the participant reclined on a chair and free to close their eyes and relax or fall asleep. Many fell asleep throughout the duration of the testing period; however, participants’ wakefulness was not recorded.

#### ABR Procedure and Analysis •

Participants were presented with 100-μsec alternating polarity clicks at a level of 100 dB peak equivalent sound pressure level (SPL) and at a rate of 11.1 per second using ER-3A insert headphones. Online filtering was applied with a high-pass filter at 100 Hz and a low-pass filter at 3000 Hz. A vertical electrode montage was used, with an active electrode at the high forehead hairline (Fz), a reference electrode at the right mastoid, and a ground at low forehead (Fpz). Impedances were maintained below 5 k Ω. ABR waveforms were averaged across 8000 presentations of each polarity.

Absolute latencies and amplitudes for waves I, III, and V of the ABR for each participant were computed online using the computer cursor. Recordings were exported to text files and ABR waveforms were plotted within a 0 to 10 msec time window by a MATLAB script. For each participant, the peaks of waves I, III, and V were chosen by the first author and were then checked a second time by an additional expert who was blind to the condition of each participant, thus providing reliability. There was no inconsistency between researchers during this selection process. Component amplitudes for waves I, III, and V were defined as the electric potential differences between peak and following trough. Absolute latencies were then used to calculate I to III, III to V, and I to V interpeak intervals. Peak-to-trough amplitudes for waves I, III, and V were used to calculate I to III, III to V, and I to V ratios.

#### FFR Procedure and Analysis •

FFR recordings took place immediately after the ABR recordings. Five amplitude-modulated stimuli were presented, which allowed the TFS and temporal envelope phase locking components to be measured simultaneously. Each stimulus consisted of three equal-amplitude pure-tone components. The central component had a frequency of 590 Hz and the two side-bands were spaced below and above this component in frequency, with spacings of 95 to 135 Hz in 10 Hz increments. Each spacing also corresponds to the amplitude modulation rate (*f*m) of the three-tone complex. The frequency components (in Hz) of the five stimuli were: 495, 590, 685; 485, 590, 695; 475, 590, 705; 465, 590, 715 and 455, 590, 725. Each component started in sine phase. Each stimulus was 200 msec in duration, including 10 msec raised-cosine onset and offset ramps. Each presentation window contained two stimuli separated by 125 msec silence. The onset polarity of the second stimulus in the pair was inverted with respect to the onset polarity of the first stimulus (Goblick & Pfeiffer 1969). The overall stimulus level was 80 dB SPL. Presentations consisting of the two stimuli were repeated at a rate of 1.5/sec. For each condition, FFR waveforms were averaged across 1500 presentations (three grand averages of 500 sweeps) of each polarity.

Stimuli were delivered using Etymotic ER30 transducers, with 6 m tubing connecting the transducers to the ear tips. This enabled the transducers to be positioned outside the experimental booth, therefore avoiding stimulus artifacts. Stimuli were presented in a random order to counteract any effects of restlessness from participants toward the end of testing. A vertical montage was used to record the FFR with an active electrode at Fz, a reference electrode at the C7, and a ground at Fpz (Krishnan & Plack 2011). Impedances were maintained below 5 kΩ. Online filtering was applied, with high pass filtering at 30 Hz, low pass filtering at 3000 Hz, and a notch filter at 50 Hz to remove mains electrical noise.

Recordings were exported to text files, read, and analyzed off-line by MATLAB scripts. Recording average responses to a direct polarity and to an inverted polarity version of each stimulus allowed the assessment of the neural representation of the temporal envelope and TFS separately. By adding the average FFRs to the direct stimulus polarity and to the inverted polarity (FFRadd), phase locking to the envelope is enhanced and phase locking to TFS is suppressed. By subtracting the FFR to the inverted stimulus polarity from the FFR to the direct stimulus polarity (FFRsub), the contribution of phase locking to the temporal envelope component is reduced and the contribution of phase locking to the TFS is enhanced (Goblick & Pfeiffer 1969). For the FFRadd, the discrete Fourier transform (DFT) at the modulation rate was calculated from the mean added responses for each stimulus condition. For the FFRsub, the DFT at the component frequencies (lower sideband, carrier frequency, and upper sideband) was calculated from the mean subtraction waveform for each stimulus condition.

To estimate the strength of the target frequency representation in the FFR relative to background noise activity, signal to noise ratios (SNRs) were calculated as the ratios between the DFT amplitude in the FFR centered at the target frequency and the average DFT amplitude across bands 5 to 33 Hz below the target frequency and 5 to 33 Hz above the target frequency. The SNRs were averaged across frequency spacing conditions and then converted to dB. For subtracted polarities, the SNR value was calculated for responses to the upper and lower sideband frequencies for each condition separately. However, to estimate an overall value for the strength of phase locking to the TFS in each condition, the average of SNRs at the carrier frequency and at the two sidebands for subtracted polarities (mean FFRsub) was taken.

To estimate the sustained latency of the envelope and TFS FFR, a MATLAB script was run to obtain a measure of group delay. The programme starts by selecting a group delay value, then calculates what phase each frequency component should have based on the group delay value selected (predicted phase). These predicted phase values are then compared against the actual phase values, after unwrapping to find the best fit. The sum of squared deviations of predicted versus observed phase values is then calculated across frequency components. To obtain the group delay final estimate, the procedure is repeated, by varying the selected group delay value, until the group delay value that minimizes the sum of squares is found. For a frequency component to be included in the group delay final calculation, a statistical criterion based on the SNR was used to determine the presence or absence of a response to the stimulus. An FFR response was accepted as present if the magnitude of the DFT at the target frequency was greater than the mean magnitude at noise frequencies surrounding it by 3 SDs of the magnitude across the noise frequencies. Noise frequencies were selected at a resolution of 2 Hz, from 5 to 33 Hz above and below the signal frequency. A group delay calculation was only included if at least three data points passed the criterion.

### Behavioral Measures

#### General Procedure •

All testing occurred in a double-walled sound-attenuating booth. Signals were created in MATLAB, and presented to the participant via Sennheiser HD 650 circum-aural headphones.

#### IPD and FDL Tests •

Using a procedure based on that described by Hopkins and Moore (2010), participants’ sensitivity to IPDs was measured for 590 Hz pure tones. This frequency was chosen as a common frequency test for the behavioral measurements for temporal coding of sounds and FFR measurements. A two-interval, two-alternative forced-choice task was used. Each interval comprised four 200 msec tones, including 10 msec raised-cosine onset and offset ramps, that were synchronous across ears. The tones were separated by 20 msec of silence within each interval and 500 msec of silence between the two intervals. In one interval, the four tones all had a zero IPD (AAAA). However, in the other interval, the second and fourth tones had a nonzero IPD (ABAB). The two intervals were randomly ordered. This form of presentation is thought to provide a clear cue for naïve listeners, and to reduce the training time required to achieve asymptotic performance (King et al. 2013). Tones were presented binaurally at 80 dB SPL.

Participants were instructed to pick the alternating interval by pressing a key (1 or 2) on a computer keyboard and were advised to focus on lateral position alternation, but that they were free to use any perceptual cue to perform the task. The response was followed by visual feedback to indicate whether the response was right or wrong. The target IPD (δ°) was initially set to 180° and could not exceed this value. A geometric adaptive two-down, one-up procedure was used. Each block of trials consisted of 16 reversals (changes in track direction). The step size was set to a factor of 2 until four reversals occurred and a factor of 1.141 for the following 12 reversals. For each block, the IPD discrimination threshold was taken as the geometric mean of δ at the last 12 reversals. Each participant completed four blocks, and the geometric mean of the last three IPD discrimination thresholds was taken as the final estimate.

FDLs were measured for the same 590 Hz pure tone used for the IPD measure. Tones were presented to the right ear at 80 dB SPL. An AAAA versus ABAB two-alternative task was used (as for IPD), with the B tones having a higher frequency than the standard 590 Hz A tones. The two intervals were randomly ordered. The procedure for estimating threshold was the same as for the IPD task, except that the percentage frequency difference between the A and B tones was varied adaptively.

#### Speech in Spatial Noise Test •

Target sentences were taken from the adaptive sentence list (ASL) corpus (MacLeod & Summerfield 1990) and the talker was a male speaker of British English. ICRA06, which represents a two-speaker background noise with two equally loud speakers of different gender (one female 3 band speech modulated noise (3bSMN) + one male 3bSMN) speaking at normal vocal effort (Dreschler et al. 2001), was used as the competing noise masker. Target speech was presented to the participants at a constant rms level of 65 dB SPL with a sampling rate of 22,050 Hz. The level of the competing talker was varied to give the appropriate SNR, except when the SNR was less than −16 dB. Below this SNR, the level of the competing talker was not increased further, but instead the level of the target speech was reduced, to prevent the combined signal becoming uncomfortably loud. In practice, this was not necessary for any of the participants. Two conditions were tested: one in which head-related impulse responses corresponding to 0°, 60°, and 300° azimuth were used for the target and two masker sentences, respectively (separated condition), and one in which the target and background speech were presented simultaneously from the front at 0° azimuth (colocated condition). Head-related impulse responses were taken from the freely available CIPIC database (Algazi et al. 2001).

Participants were asked to repeat sentences presented in a competing talker background. The background began 500 msec before the target sentence, and continued after the target sentence had finished for about 700 msec (the exact value depended on the length of the target sentence). The testing session began with a short “warm-up” period, in which two lists (which were short versions with only half the number of sentences as the full ASL lists) were presented in the separated and colocated condition, respectively. The first sentence in each list was initially presented at 12 dB SNR. After this, two consecutively presented ASL sentence lists, each made up of 30 sentences, were used for each condition. The order of presentation of conditions was counterbalanced across pairs. Unlike the first two lists, the first sentence in each of the full lists was initially presented at 10 dB SNR. The SNR of the target and competing talker has varied adaptively. If a participant identified two or more keywords correctly in a sentence, the next sentence was presented with a SNR that was *k* dB lower, and if the participant identified fewer than two keywords correctly, the next sentence was presented with a SNR that was *k* dB higher. *k* was equal to 4 dB for the first two turn points, then equal to 2 dB for the subsequent turn points. The adaptive track continued until the 30 sentences were presented. For each sentence list, the total number of keywords presented at each SNR was recorded, as well as the number of keywords that were identified correctly for each SNR.

For each SNR, the total keywords presented and keywords correct were summed for the two sentences lists that were presented for each condition (Hopkins & Moore 2009). These values were used to perform a probit analysis (Finney 1971), from which the SNR required for 50% correct identification was estimated for each participant and each condition. For each condition, the mean of the estimated two SNR values, required for 50% correct identification for the two used sentence lists, was taken as the final estimate (the SNRs for the two short lists were not included in the final estimate). Spatial release from masking (SRM; Plomp & Mimpen 1981; Hawley et al. 1999) was measured by calculating the difference between the SNR for 50% correct in the colocated condition and the SNR for 50% correct in the separated condition.

### Self-Report of Auditory Disability Measures

Participants’ self-report ability to hear in different everyday situations was measured on their first session, before assessing their hearing ability using PTA. This was done to not bias the self-report results. The original 49-item version of the SSQ (Gatehouse & Noble 2004) was administrated for the present study. The 49 items were related to three subscales, with 14 items assessing an individual’s ability to detect and understand speech in a variety of competing contexts (Speech subscale), 17 items assessing spatial listening abilities (Spatial subscale), and 18 items assessing qualities of hearing including ease of listening, naturalness, and clarity of sounds (Qualities subscale).

Most of the participants (n = 44) completed the SSQ questionnaire in an interview format in a quiet room. The researcher read the questions aloud, and participants were asked to respond to each item, by marking a number, rating themselves with a score on a scale ranging from 0 (not able at all, complete absence of a quality or total need for effort) to 10 (perfectly able to, complete presence of a quality or complete absence of the need for effort). Singh and Kathleen Pichora-Fuller (2010) found minimal differences in mean SSQ scores when the questionnaire was given in an interview format or completed at home and returned by mail. Therefore, participants were given the option to complete by either method. Only 16 participants (nine controls and seven T1DM) chose to complete the questionnaire on their own. Those received the questionnaire form together with the participant information sheet and returned it on their first session.

### Statistical Analyses

All statistical analyses were carried out with SPSS (IBM statistics SPSS version 22). If the difference between the paired values of a measure was normally distributed, paired samples *t* tests were run. However, when the difference was not normal, and could not be normalized using transformation algorithms, a nonparametric Wilcoxon signed-ranks test was used. Correlation coefficients, Pearson’s (*r*) or Spearman’s (*rs*) for nonnormally distributed variables, were calculated to assess the relations between measures. Bonferroni correction was used to control for multiple comparisons within each research question.

## RESULTS

### Electrophysiology

**Fig. 2.**
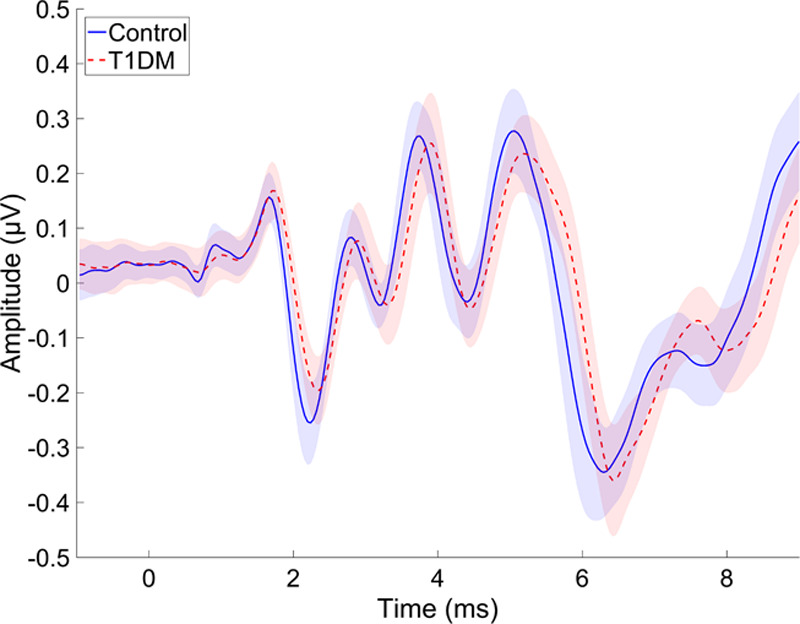
Grand average auditory brainstem response waveforms plotted for the control and T1DM groups (n = 30 in each group). The solid line shows the mean response across individuals and the shaded area shows 95% confidence intervals calculated for each time point. T1DM indicates type 1 diabetes mellitus.

**Fig. 3.**
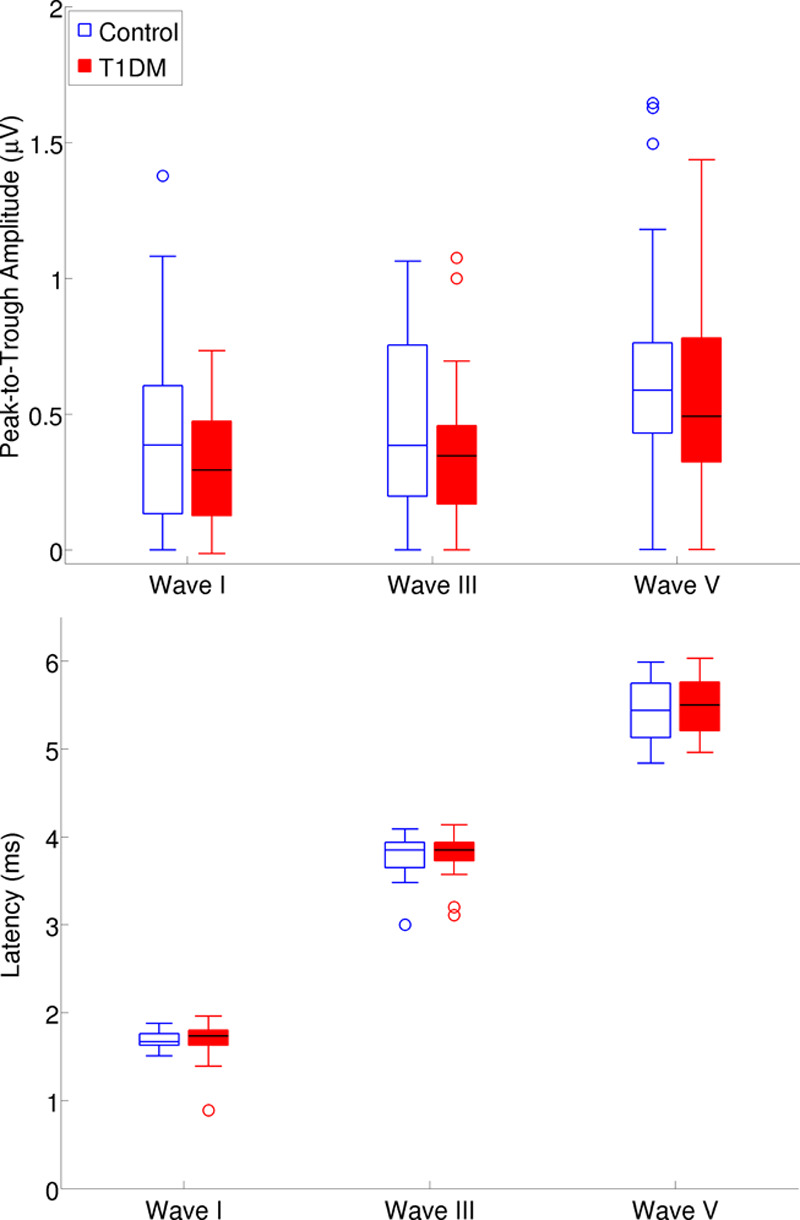
Peak-to-trough amplitudes (upper panel) and latencies (lower panel) for auditory brainstem response waves I, III, and V. The rectangle shows the interquartile range. For this and subsequent plots, the bold lines inside rectangles show the median, and whiskers show the maximum and minimum values excluding outliers. Open circles show outliers defined as 1.5 × interquartile range (IQR) or more above the third quartile or 1.5 × IQR or more below the first quartile. T1DM indicates type 1 diabetes mellitus.

Figure 2 shows the grand average ABR waveforms plotted for the control and the T1DM groups. Figure 3 shows wave I, III, and V peak-to-trough amplitudes (upper panel) and absolute latencies (lower panel) for the two groups. The difference between the two groups was not significant for any of the ABR amplitude or latency measures (see Table in Supplemental Digital Content 3, http://links.lww.com/EANDH/A563, which shows the statistics for all variables used in the analyses on the ABR data).

**Fig. 4.**
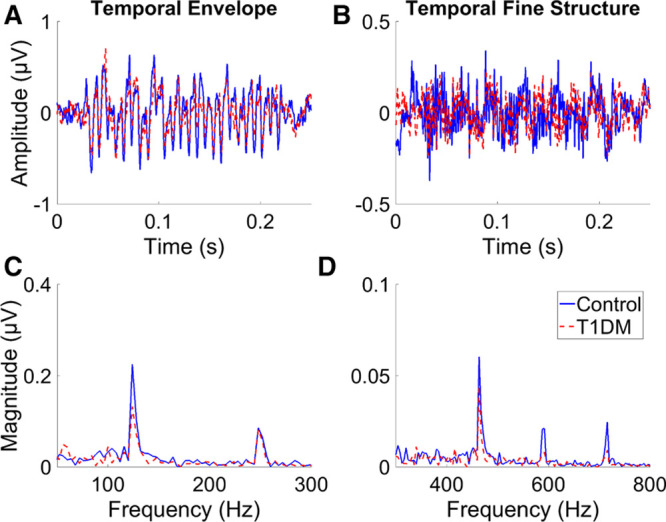
Average waveforms and spectra of the frequency-following response for the stimulus with frequency components 475, 590, and 705 Hz for the control and T1DM groups. A, The addition waveform reflecting phase locking to the temporal envelope. B, The subtraction waveform reflecting phase locking to the temporal fine structure. C, The spectrum of the addition waveform. D, The spectrum of the subtraction waveform. T1DM indicates type 1 diabetes mellitus.

Figure 4 shows the average added (A) and subtracted (B) waveforms of the FFR for one of the five stimuli (475,590,705 Hz). Figure 4 also shows the average added (C) and subtracted (D) spectra. Spectral peaks can clearly be seen corresponding to the modulation frequency in the addition spectra, and to the component pure-tone frequencies in the subtraction spectra. The FFRs for the control group are larger than those for the T1DM group.

**Fig. 5.**
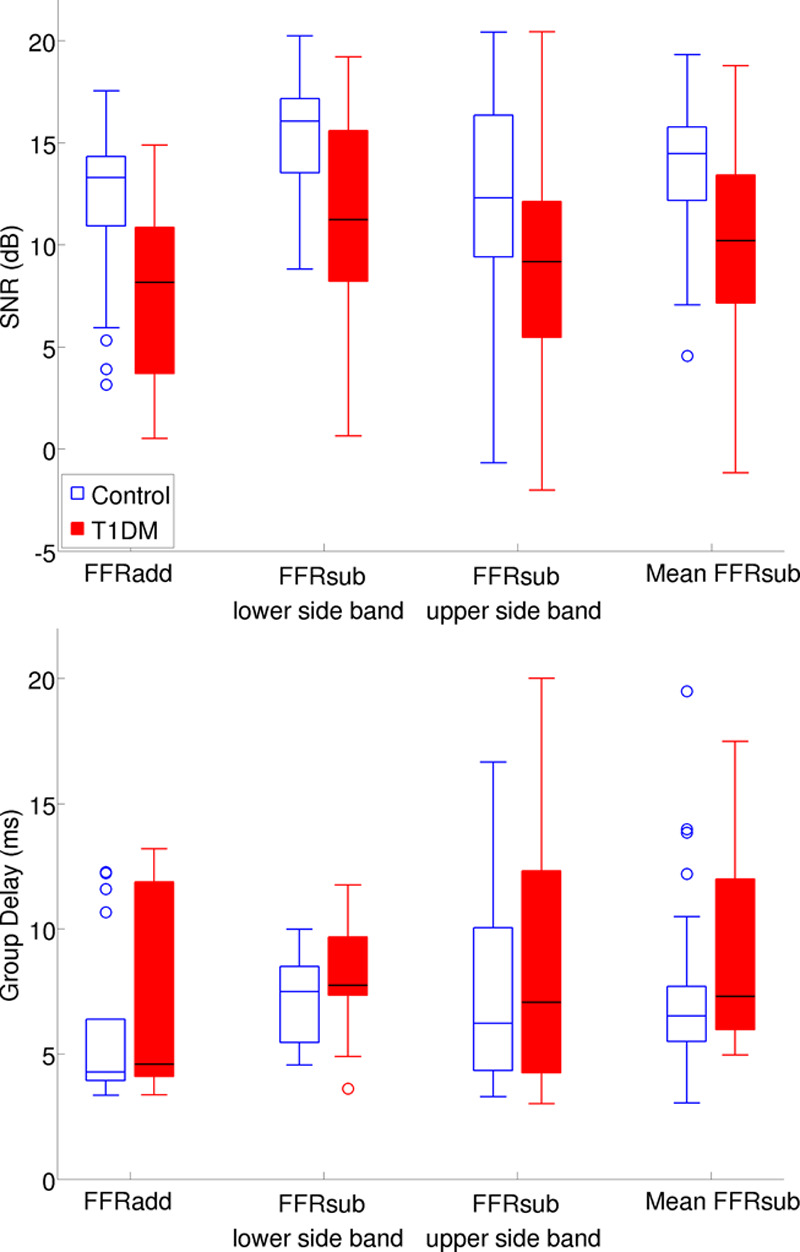
FFR Signal to noise ratios (SNRs) and group delays for the different measures. Upper panel, SNR for the addition waveform (FFRadd), the lower sideband subtraction waveform (FFRsub lower sideband), the upper sideband subtraction waveform (FFRsub upper sideband), and the mean subtraction waveform (mean FFRsub). Lower panel, Group delays for FFRadd (N = 17), FFRsub lower sideband (N = 22), FFRsub upper sideband (N = 17), and mean FFRsub (N = 29). FFR indicates frequencyfollowing response; T1DM, type 1 diabetes mellitus.

Figure 5 shows FFR SNRs and group delays for the different measures. Only a proportion of the matched pairs had values for each group delay measure that passed the SNR criteria. The number of T1DM participants with available group delay values was 18 for FFRadd and 29 for FFRsub. The number of control participants with available group delay values was 27 for FFRadd and 30 for mean FFRsub. Thus, the number of group delay values for FFRadd was substantially smaller for the T1DM group than for the control group. The number of matched pairs available for the analysis was 17 for FFRadd and 29 for mean FFRsub.

After applying a Bonferroni correction (*α* = 0.0063), the difference between the two groups was significant for all the SNR values for FFRadd, FFRsub lower sideband, FFRsub upper sideband, and mean FFRsub (Table 1). However, none of the group delay values was significantly different between the two groups (see Table in Supplemental Digital Content 4, http://links.lww.com/EANDH/A564, which shows the statistics for all variables used in the analyses on the FFR group delay data).

**TABLE 1.**
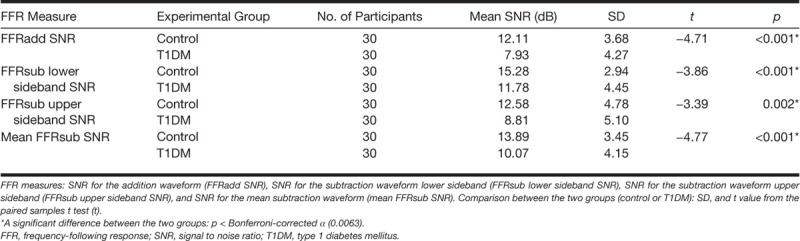
Statistics for FFR SNR group comparisons

#### Relations Between Amplitude or Latency Measures of ABR and FFR •

In Bonferroni-corrected correlations (*α* = 0.0063), a significant correlation was observed between group delay for FFRadd and ABR wave V absolute latency in the T1DM group (n = 18, *rs* = 0.63, *p* = 0.005). However, this correlation was not significant in the control group. No significant correlation was found between SNRs for FFRadd or mean FFRsub and wave V peak-to-trough amplitudes for the ABR, for either the control or T1DM groups.

### Behavioral Measures

**Fig. 6.**
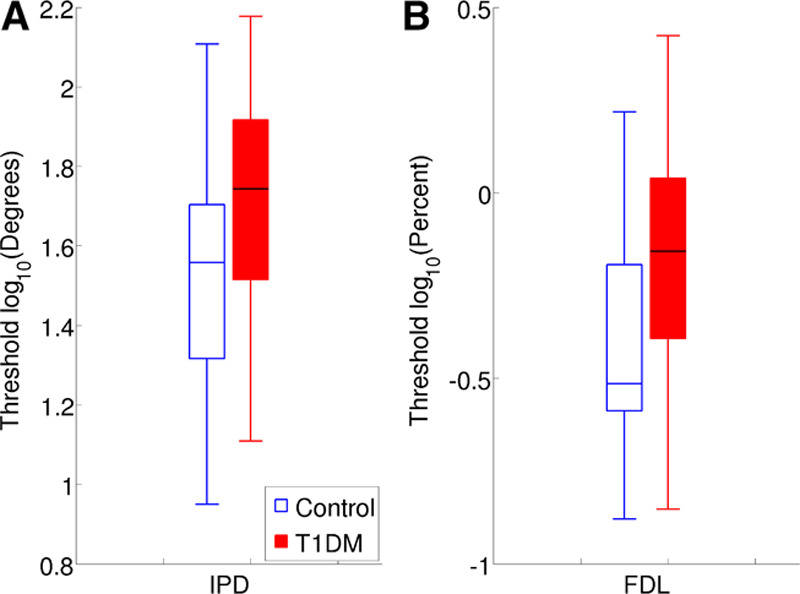
log-transformed interaural phase difference (IPD) thresholds and log-transformed frequency difference limens (FDLs) for the control and the T1DM groups. A, Interaural phase difference thresholds (IPD). B, Frequency difference.

Figure 6 shows the log-transformed IPD thresholds and log-transformed FDLs for the control and the T1DM groups. In a Bonferroni-corrected paired *t* test (*α* = 0.01), log-transformed IPD thresholds and log-transformed FDLs were both significantly higher for the T1DM group than for the controls (Table 2).

**TABLE 2.**
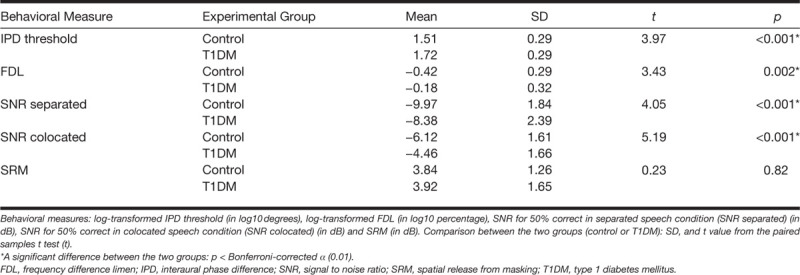
Statistics for the behavioral group comparisons

**Fig. 7.**
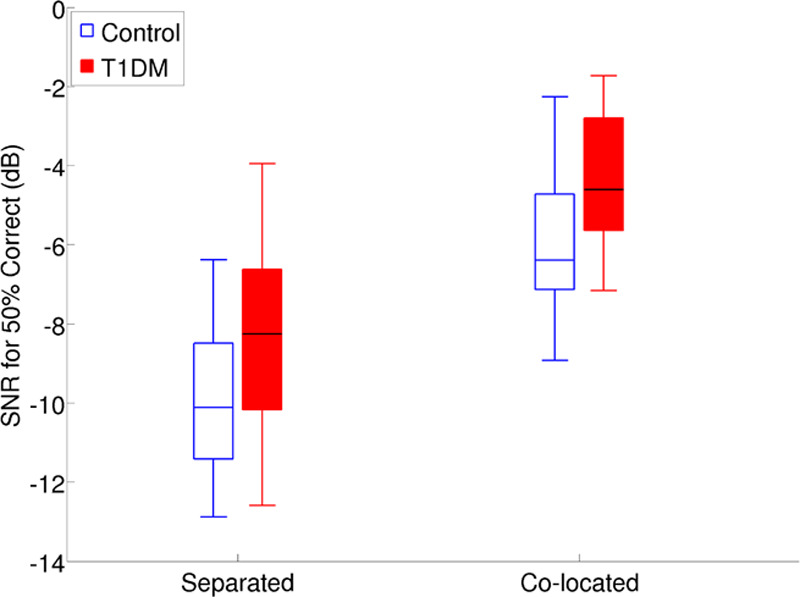
Signal to noise ratios (SNR) for 50% correct for the separated and colocated speech-in-noise conditions. T1DM indicates type 1 diabetes mellitus.

Figure 7 shows the SNR for 50% correct for the control and T1DM groups for the separated and colocated speech conditions. There was a significant difference between the two groups after Bonferroni correction in both conditions (Table 2). However, there was no significant group difference in SRM.

#### Relations Between the Behavioral Measures •

Log-transformed IPD thresholds were strongly correlated with log-transformed FDLs in the control and T1DM groups (*r* = 0.70, *p* < 0.001; and *r* = 0.60, *p* < 0.001, respectively). A strong correlation was also observed between the SNR for 50% correct in the separated and in the colocated condition in the control and T1DM groups (*r* = 0.74, *p* < 0.001; and *r* = 0.73, *p* < 0.001, respectively). The correlation between log-transformed FDLs and SNRs for 50% correct in the separated condition for the T1DM group did not remain significant after the correction (*r* = 0.47, *p* = 0.02; *α* = 0.006). There were no other significant correlations between FDLs or IPD thresholds and speech-in-noise measures.

### Self-Report of Auditory Disability Measures

**Fig. 8.**
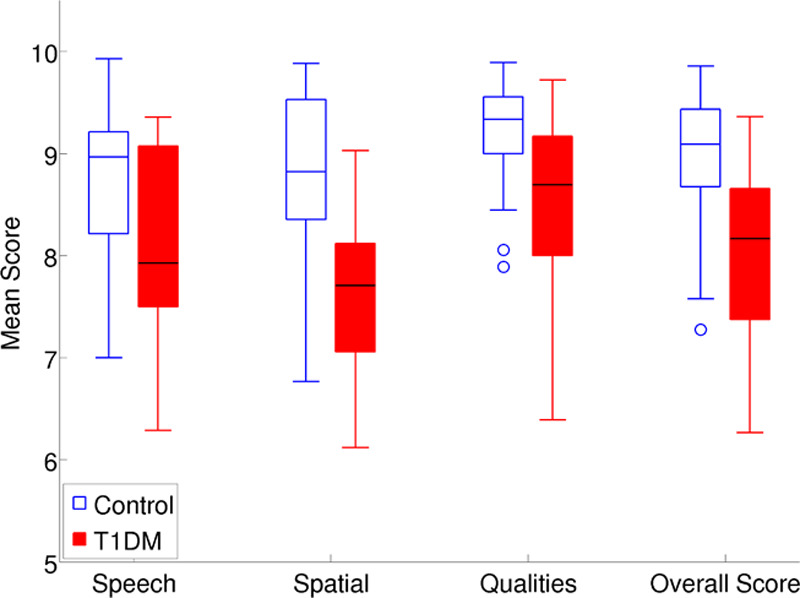
The Speech, Spatial and Qualities (SSQ) subscale scores and the overall SSQ scores. T1DM indicates type 1 diabetes mellitus.

Figure 8 shows the SSQ subscale scores, and the overall SSQ scores, for the control and T1DM groups. An analysis of variance revealed significant main effects of group and SSQ subscale [*F* (1, 58) = 24.04, *p* < 0.001; *F* (2, 12) = 26.74, *p* < 0.001, respectively], and there was also a significant interaction between group and SSQ subscale [*F* (2, 12) = 4.07, *p* < 0.02]. In Bonferroni-corrected paired *t* tests (*α* = 0.013), the T1DM group showed significantly lower scores than the control group on each of the SSQ subscales. The T1DM group had significantly lower overall SSQ scores than the control group (Table 3).

**TABLE 3.**
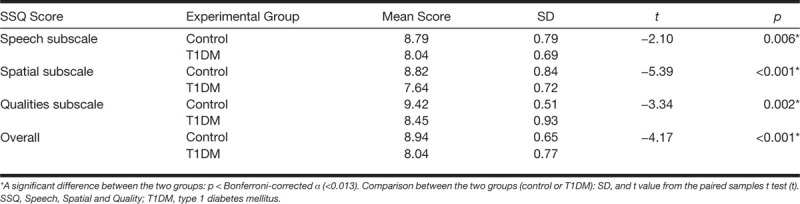
Statistics for the SSQ Subscale Scores and the Overall SSQ Scores Group Comparisons

### Relations Between the Experimental Measures and the Effects of DM-Related Factors

The primary focus of this study was to determine whether T1DM affects neural coding of the rapid temporal fluctuations of sounds, and how any deficits may impact on behavioral performance, and not on the relations between experimental measures. Because there was a significant difference between the two groups in most of the measures, these significant measures also correlate across the whole cohort. For the present analysis, groups were analyzed separately when investigating the relations between the experimental measures and only statistically significant correlations following Bonferroni correction are reported and discussed.

#### Relations Between Experimental Measures •

Neither ABR wave I nor wave V peak-to-trough amplitudes nor absolute latencies correlated significantly with any of the behavioral measures, for either the control or T1DM groups (see Table in Supplemental Digital Content 5, http://links.lww.com/EANDH/A565). Nor was there a significant relation between FFRadd, mean FFRsub SNRs nor FFR group delay values and any of the behavioral measures, for either the control or the T1DM groups (see Table in Supplemental Digital Content 5, http://links.lww.com/EANDH/A565). One weak correlation was observed between FFRadd SNRs and log-transformed IPD thresholds in the T1DM group. However, this correlation did not remain significant after the correction (*α* = 0.0031). In Bonferroni-corrected correlations, for the T1DM group, there was a significant correlation between wave I latency and log-transformed FDLs (*r* = 0.85, *p* < 0.001), but no significant correlation between wave I latency and log-transformed IPD threshold.

No correlation remained significant, following a Bonferroni correction (*α* = 0.0063), between overall SSQ scores and ABR or FFR amplitude and latency measures, for either the control group or for the T1DM group. However, there was a strong correlation between overall SSQ scores and SNRs in the separated speech condition, for the T1DM group (*r* = −0.48, *p* = 0.008).

#### The Effects of DM-Related Factors •

**Fig. 9.**
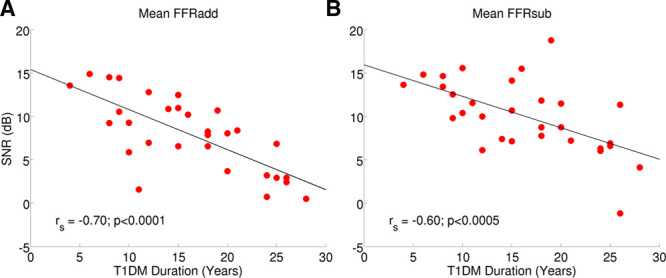
T1DM duration plotted as a function of (A) the addition waveform (FFRadd) and (B) mean subtraction waveform (FFRsub) signal to noise ratios (SNR). Spearman correlation coefficients are reported, with associated p-values. FFR indicates frequency-following response; T1DM, type 1 diabetes mellitus.

After Bonferroni correction (*α* = 0.0063), FFRadd and mean FFRsub SNRs correlated significantly with DM duration (*_r_s* = −0.7, *p* < 0.001, *_r_s* = −0.6, *p* = 0.005, respectively, Fig. 9). None of the other measures correlated significantly with DM duration. Independent-samples *t* tests showed no significant difference between T1DM participants with clinically diagnosed neuropathy or retinopathy and those without, for any of the experimental measures.

## DISCUSSION

### Do T1DM Patients Show Evidence of Cochlear Neuropathy or Central Neural Dysfunction?

#### Auditory Brainstem Response •

In the present study, the amplitudes and absolute latencies for ABR wave I were similar across the two groups, showing no evidence of cochlear neuropathy. These results are in keeping with those of Rance et al. (2014), who found that peripheral auditory function in listeners with T1DM was normal, with distortion-product otoacoustic emissions (DPOAEs) present in each ear, indicating normal cochlear function, and that absolute latencies and amplitudes for wave I of the click-evoked ABR were equivalent to the age- and sex-matched controls. It is known that high-frequency hearing loss as a result of damage to the basal segments of the cochlea can cause a delay in wave I with no effect on wave V latency, making the wave I to V interval shorter (Coats & Martin 1977). However, in the present study, PTA thresholds at 6 and 8 kHz were similar across the two groups.

No significant differences were found between the control and T1DM groups in peak-to-trough amplitudes or absolute latencies of waves III and V nor were any significant differences found between the two groups in peak-to-trough amplitude ratios or interpeak intervals for I to III, III to V, and I to V. Thus, the present ABR data provide no clear evidence of reduced conduction efficiency, which may result from demyelination, nor of neural dyssynchrony, another possible consequence of demyelination or axonopathy, in T1DM patients in the absence of an elevation in audiometric threshold. The results of this study are in contradiction with those of studies which have found some differences between the ABR waveforms of DM patients and controls (Parving et al. 1990; Bayazit et al. 2000; Lisowska et al. 2001; Frisina et al. 2006; Rance et al. 2014). A possible explanation for the discrepancy between the present results and previous findings is that the T1DM and healthy controls in the present study were closely PTA-matched, whereas DM PTA thresholds in previous studies were always elevated when compared with those of the controls, even in studies where DM average hearing levels were within normal or near-normal ranges (Rance et al. 2014, 2016). It is also possible that if a higher stimulus presentation rate had been used in the present study, ABR waveforms would have been more strongly affected by T1DM, as reported by Rance et al. (2014). They found the mean maximum rate with a recordable ABR for the T1DM group to be significantly lower than for the control group and concluded that the abnormal ABRs to high rate stimuli suggest that the neural systems of T1DM patients are more easily stressed compared with controls, consistent with the results in other neuropathologies such as multiple sclerosis (Fowler & Noffsinger 1983).

#### Frequency-Following Response •

The FFR SNRs for added polarities (envelope) as well as for the subtracted polarities (TFS) were significantly and substantially lower in the T1DM group compared with the age-, sex-, and PTA-matched healthy controls. The reduced SNRs in T1DM patients suggest that the capability to phase lock to stimuli may be impaired as a result of neuropathy of the auditory pathway up to and including the rostral brainstem.

Similar to ABR latency results, the FFR group delay data provide little evidence that T1DM affects neural conduction time: no significant differences in group delay for responses to FFRadd and FFRsub were found between the T1DM and control groups, although there was a trend for prolonged group delay for FFRadd and FFRsub in the T1DM group. These results suggest either that ABR and FFR latencies are not sensitive to timing changes in the brainstem associated with T1DM or that these changes are slight in young normal-hearing T1DM patients.

#### Relations Between ABR and FFR Amplitude and Latency Measures •

It has been claimed that the FFR has similar neural generators to wave V of the ABR, that is, the inferior colliculus (Smith et al. 1975; Daly et al. 1976; Stillman et al. 1976). However, the evidence is inconclusive (Gardi et al. 1979; Batra et al. 1986; Kuwada et al. 1986; Dolphin & Mountain 1992; Purcell et al. 2004). A poor correlation between ABR and FFR latencies was also reported when ABR and FFR were directly compared by Hoormann et al. (1992), suggesting multiple generators of the FFR, or that the FFR may have separate but also overlapping generators to the ABR (Stillman et al. 1978; Gardi et al. 1979; Davis & Britt 1984; Batra et al. 1986; Bidelman 2015). Moreover, using magnetoencephalography, a recent study by Coffey et al. (2016) reported cortical contributions to the FFR in humans.

In the present data, the FFRs to the envelope and the TFS were found to occur significantly later than wave V of the ABR. The only significant correlation was observed between group delay for the FFR to the envelope and ABR wave V absolute latency in the T1DM group. No strong conclusions can be drawn, due to the small sample size (n = 18) and the fact that this correlation was not significant in the control group (n = 27). In addition, neither of the amplitudes for these FFR components was found to correlate with the amplitude of ABR wave V.

The results of the present study support earlier findings suggesting separate neural generators for the FFR and wave V (Hoormann et al. 1992) and indicating a separate processing component within the auditory brainstem that is unique to more complex stimuli (Song et al. 2006). These results may explain why T1DM participants in this study demonstrated a normal wave V latency and amplitude in the presence of a disordered FFR. It could be that DM-associated damage to parts of the auditory brainstem responsible for generating all or part of the continuous FFR does not affect its ability to generate wave V of the ABR.

The present study suggests that the FFR may be more sensitive to subtle auditory processing deficits in T1DM patients than the ABR, and thus can identify deficits that may be missed if only the conventional click-evoked ABR is performed. The amplitude-modulated complex tones used to elicit the FFR may better represent the complex acoustic signals of speech (Shannon et al. 1995; Alcántara et al. 2012) than a click stimulus that lacks frequency specificity and ecological validity. The use of a more complex stimulus to assess the auditory brainstem function in T1DM patients could reveal temporal processing deficits to which the click-evoked ABR may not be sensitive. However, although these results suggest that the FFR could have clinical potential as a diagnostic test to identify AN and brainstem neural processing deficits in patients with T1DM, measurement of the FFR has not yet proven to be sufficiently fast or reliable to rival a measurement such as the ABR. Future studies are required to determine the neural generators and to establish normative latency values for the FFR, as well as to further understand the relation between ABR and FFR measures.

### Is T1DM Associated With Poorer Performance on Behavioral Tasks, in the Absence of an Elevation in Audiometric Threshold?

T1DM patients in this study showed evidence of deficits in IPD sensitivity and frequency discrimination. These findings suggest an association between T1DM and deterioration in temporal processing abilities in the presence of normal-hearing detection levels, providing support for the conclusion of Rance et al. (2014) that temporal processing abilities deteriorate in normal-hearing T1DM patients, as evidenced by impaired perception of rapid amplitude modulation.

The present data also provide evidence of significantly impaired speech-in-noise performance in T1DM patients in the absence of an elevation in PTA thresholds, in keeping with previous speech audiometry research on normal-hearing DM patients (Kakarlapudi et al. 2003; Rance et al. 2014). As expected, in the present study, the T1DM group showed significantly higher (worse) SNRs than the healthy controls in separated and colocated conditions. However, mean SRM values for the two groups were equivalent: the difference between two groups in separated and colocated conditions was roughly equal. This finding does not support the hypothesis that T1DM patients would have lower SRM values than those of the healthy controls due to a decline in temporal coding. The results are in contrast with those of Rance et al. (2016), who found speech reception thresholds for children with T1DM to be significantly higher than the sex- and age-matched controls in the separated condition, where binaural difference cues were available, whereas mean reception thresholds for the two groups were equivalent when no binaural cues were available (colocated condition). Again, a possible explanation for the discrepancy between the present results and the findings of Rance et al. is the elevated PTA thresholds of their DM patients compared with those of the controls, whereas in the present study, the DM and healthy controls were closely PTA-matched.

The current results provide no evidence of a specific “binaural disadvantage” for DM participants and suggest that speech perception difficulties in T1DM patients are more general deficits, possibly a combination of deficits in general temporal processing and neural coding, including frequency selectivity or intensity coding, as well as DM-related nonsensory cognitive deficits, which could affect auditory processing ability, such as attention (Ryan et al. 1993; Rovet & Alvarez 1997) and memory (Biessels et al. 1994).

### Is T1DM Associated With Self-Report of Auditory Disability in the Absence of an Elevation in Audiometric Threshold?

Mean scores on the SSQ were generally quite high for both groups, with the control group scoring higher than 8.7 points and the T1DM group scoring higher than 7.6 points for the mean overall SSQ score and mean SSQ subscale scores. The mean scores of the control group on all three subscales fall within the normal range established by Banh et al. (2012) for the best scores that could reasonably be expected from healthy young adults who have audiometric thresholds within normal limits, that is, thresholds that are considered clinically normal in most or all of the speech range, and are not likely to be candidates for hearing aids. For Banh et al., in normal-hearing young adults, the mean overall SSQ and the SSQ subscale scores were 8.8, 8.5, 8.6, and 9.4 points, respectively.

In the present study, the T1DM group had significantly lower overall SSQ scores and consistently reported significantly more difficulties than the control group on the SSQ subscales. Different patterns of results across the subscales were observed in the two groups. Both groups reported having the least disability on items from the Qualities subscale, but whereas the control group had roughly equal mean scores on the Speech and Spatial subscales, the T1DM group reported the greatest disability on items from the Spatial subscale. This was evidenced by the significant interaction observed between group and SSQ subscale, which probably was driven by the T1DM group’s relatively low scores on the Spatial subscale. In keeping with the results of Rance et al. (2016), the present study provides evidence that T1DM is associated with self-report of auditory disability in the absence of an elevation in audiometric threshold.

### Relations Between Experimental Measures and the Effects of DM-Related Factors

#### Relations Between Electrophysiological and Behavioral Measures •

Only ABR wave I latency, in the T1DM group, was negatively correlated with the FDL. No other correlations were found between the amplitudes and latencies of waves I and V and the behavioral measures in the healthy control and T1DM groups considered independently. The present data also show no link between the synchronization strength and group delay latency of the FFR and the behavioral measures when the groups were considered independently (although there were, unsurprisingly, strong correlations across the whole cohort between these measures as they were all affected by DM).

The finding that the FFR did not correlate with FDLs for either group considered independently is in keeping with Clinard et al. (2010), who, using pure-tone stimuli, did not observe a correlation between FFR measures and FDLs in normal-hearing listeners. However, this is contrary to other observations (Marmel et al. 2013; Xu & Gong 2014) of a negative correlation between FFR magnitude and FDL measures of temporal coding (higher FFR related to better performance).

The absence of significant correlations in the present study means that one should be cautious about concluding that the neural deficits observed were in some way causally linked to the behavioral deficits. However, this remains a possibility, despite these negative findings.

#### Relation Between Self-Report of Auditory Disability and Electrophysiological and Behavioral Measures •

There was a strong correlation between overall SSQ score and SNR in the separated speech condition, for the T1DM group. The pattern of these correlations points to some degree of binaural deficits in DM participants, possibly due to their reduced sensitivity to TFS information, supporting the hypothesis that binaural deficits underlie the self-reported deficits in T1DM. However, the overall results are equivocal, taking into consideration the contradictory evidence reported above that no significant difference was found between the control and T1DM groups in SRM, while the difference in SNRs between the two groups was roughly equal in separated and colocated conditions.

#### Effects of DM-Related Factors •

DM participants with the longest DM duration displayed the lowest FFR SNRs for responses to both the envelope and TFS. This suggests that the FFR is sensitive to auditory processing deficits which ensue from subtle vascular, metabolic, or endocrine derangements, associated with T1DM, although DM duration did not correlate significantly with any of the other measures. Strong correlations between DM duration and hearing deficits in DM patients have been reported (Taylor & Irwin 1978; Parving et al. 1990; Virtaniemi et al. 1994). However, others have not observed such effects in longer-lasting DM (Ottaviani et al. 2002; Dąbrowski et al. 2011).

The present data provide no evidence that patients with diabetic neuropathy or retinopathy are more likely to present with neuropathic complications involving the AN and central auditory pathways: no correlation was found between the presence of neuropathy or retinopathy and greater hearing deficits. These findings are in keeping with Lisowska et al. (2001) and Tay et al. (1995), and in contrast with those of Virtaniemi et al. (1994), Bayazit et al. (2000), and Rance et al. (2014).

The lack of correlation in our study between hearing deficits and the presence of retinopathy and neuropathy may in part be explained by: (1) a lack of power in the present study; (2) by the use of self-report to determine whether or not each DM participant had diagnosed clinical neuropathy or retinopathy, making the findings unreliable. Moreover, the majority of our DM participants (especially those following up with general practitioners rather than specialized DM centers) reported that they had not undergone neurological exams for over a year. For this reason, a short questionnaire was used to take relevant DM-related history from all DM participants, while each participant with no confirmed clinical neuropathy diagnosis was also screened for the absence or presence of typical neuropathy symptoms such as numbness, shooting pain, and burning pain. Thirteen of the 24 DM participants with no clinically diagnosed neuropathy confirmed the presence of one or more typical neuropathy symptoms. Thus, there is a possibility that some of those patients actually had the condition but had not been diagnosed. So far, only Rance et al. (2014) and colleagues appear to have performed all necessary measurements confirming the presence of diabetic neuropathy in six out of 10 subjects with T1DM in their study population. They found auditory dysfunction to be correlated with both visual acuity and degree of somatic peripheral neuropathy.

### Are the DM-Related Deficits Due to Peripheral or Central Auditory Processing Deficits?

Pathological and clinical studies of DM-related auditory dysfunction in both animals and humans have been inconclusive in determining the underlying causes or whether there is a pattern of pathological deterioration. Hence, the site of lesion in DM-related auditory dysfunction is still strongly contested. Various studies have reported different effects on anatomical structures and have proposed causes such as: interference of nutrient transportation due to a thickening in the vessels of the basilar membrane, oxidative stress—that is, the excessive production of reactive oxygen species from electron leakage in the mitochondria caused by the hyperglycemic state, resulting in neuronal cell death (Akinpelu et al. 2014b), atrophy of spiral ganglion neurons, demyelination of the AN, and the loss of outer hair cells or inner hair cells (Makishima & Tanaka 1971; Fukushima et al. 2006; Kariya et al. 2010).

These pathological changes and metabolic disturbances can result in peripheral (cochlear), central auditory pathway, or combined peripheral and central deficits. The findings of previous research on auditory function in patients with T1DM are highly contradictory. For example, Ottaviani et al. (2002) report cochlear dysfunction, as measured by OAEs, in normal-hearing T1DM patients and Lisowska et al. (2001) report peripheral and central auditory dysfunctions, as measured by DPOAEs and ABRs, in normal-hearing T1DM patients, whereas normal-hearing T1DM patients in the Rance et al. (2014) study who showed evidence of central auditory pathway abnormality had DPOAEs present in each ear, indicating normal cochlear function, and absolute latencies and amplitudes for wave I of the click-evoked ABR equivalent to the age- and sex-matched controls.

The present data are consistent with the findings of Rance et al. (2014) showing no evidence for cochlear neuropathy in the T1DM group. In the present study, absolute latencies and amplitudes for wave I of the click-evoked ABR were similar to those for the age-, sex-, and PTA-matched healthy controls, whereas the rest of the results provide substantial evidence for DM-related central auditory deficits; these include reduced FFR responses, higher IPD and FDL thresholds, and worse speech-in-noise performance. In terms of identifying a site of lesion, the FFRsub results are most specific. Phase locking to TFS largely disappears moving upward through the auditory pathway, with the upper limit of phase locking reducing to 250 Hz or lower at the level of the primary auditory cortex (Wallace et al. 2002). Lower SNRs for the subtracted polarities (TFS) in the T1DM group suggest the presence of a lesion either in the rostral brainstem or earlier in the auditory pathway. It should be noted that a limitation of the present study was that OAEs were not measured. It is possible that OAE measures would have revealed cochlear dysfunction not revealed by PTA.

A possible explanation for greater DM-related effects being evident using central measures such as FFR, rather than peripheral measures such as PTA, OAEs, and wave I of the ABR, is that the auditory pathway can be thought of as comprising several processing stages, each of which may be affected by relatively subtle alterations, for example, a certain percentage of neural loss. The initial effects of DM at each stage may be small, but the cumulative effects will increase with each additional stage reached. Thus, it may be speculated that if the neural response is reduced at each stage of the pathway, albeit by only a small percentage, then by the time the bottom-up input from the cochlea has passed several stages, the response may have decreased significantly.

#### Limitations •

Although the present study corrected for multiple comparisons within each main outcome measure category, a more conservative approach would be correct across all of the outcome measures. When this was done across all 29 group comparisons (*α* = 0.0017), most of the significant comparisons remained significant, although a few comparisons (FFRsub upper sideband SNR, FDL, SSQ Speech subscale, and SSQ Qualities subscale) did not survive correction with this conservative criterion. Hence a future, more focused, validation study would be useful to confirm that these measures are associated with T1DM.

Moreover, although T1DM is not typically associated with reduced intelligence, subtle neurocognitive impairments were reported in children (Ryan et al. 1990; Rovet & Alvarez 1997; Ryan 1999; Schoenle et al. 2002) and adults (Bale 1973; Skenazy & Bigler 1984; Ryan & Williams 1993) with T1DM. The frequent transient alterations of blood glucose levels which DM patients experience have been found to affect attentional abilities in children (Ryan et al. 1990; Davis et al. 1996) and adults with DM (Holmes et al. 1983; Widom & Simonson 1990), as well as in nondiabetic healthy participants (Stevens et al. 1989; McCrimmon et al. 1996). Poorer attention has been reported in adults with longstanding DM (Bale 1973; Ryan & Williams 1993) and has been related to chronic hyperglycemia, duration of DM (Ryan et al. 1993), and recurrent severe hypoglycemia (Skenazy & Bigler 1984; Langan et al. 1991; McCrimmon et al. 1996). A meta-analysis by Brands et al. (2005) provided evidence of significantly lowered cognitive performance in the T1DM patients compared with nondiabetic healthy controls. The pattern of their findings does not support an overall impairment of cognitive abilities in T1DM patients, but rather mild to moderate deficits resulting in a slowing of mental processing and diminished mental flexibility. The authors report that lowered cognitive performance seemed to be associated with the presence of microvascular complications but not with hypoglycemic episodes or poor metabolic control.

The majority of the T1DM group in the present study, especially those with longer DM duration, were diagnosed when they were children. Children with T1DM are at greater risk of frequent high and low blood glucose excursions, recurrent episodes of acute hypoglycemia and hypoglycemic seizures. These factors have been related to subtle impairment of cognitive functions (Golden et al. 1989; Rovet & Ehrlich 1999; Ryan 1999; Schoenle et al. 2002). Hence, it is possible that multiple aspects of cognitive functioning may have been disrupted in the present study’s young, normal-hearing T1DM group, which may have affected performance on the behavioral tasks in the study. The present study did not assess whether there had been a history of severe episodes of hypoglycemia or hypoglycemic seizures among the DM patients. Moreover, participation in the study was quite time consuming and may have been associated with fatigue. Although this was minimized through the taking of regular breaks with the provision of refreshments suitable for DM patients, no measurement of blood glucose was taken to confirm the absence of hypoglycemia. Future study is strongly encouraged to understand further the mechanisms that underlie the auditory deficits in T1DM patients. Such research should use diagnosis confirmed through neurological assessment, to explore whether the presence of neuropathy or of retinopathy are risk factors for AN and central auditory pathway involvement in patients with T1DM. Cognitive studies which carefully review T1DM patients’ medical history are also required to investigate the potential impact of cognitive problems and of individual differences in cognitive functioning on understanding speech-in-noise in patients with T1DM.

## CONCLUSIONS

The main conclusions drawn from this study can be summarized as follows:

1. Despite clinically normal-hearing detection levels as measured by PTA, clear neural deficits are seen in T1DM patients, evidenced by reduced synchrony to the temporal envelope and TFS in the FFR, and by elevated IPD thresholds and FDLs.
2. T1DM is associated with deficits in real-world hearing ability, including speech-in-noise perception and self-reported ability. However, nonauditory deficits associated with T1DM, including cognitive deficits, may contribute to variability in real-world performance.
3. The results suggest strongly that PTA is not fit for purpose as a measure of the underlying hearing dysfunction in T1DM patients. The FFR may provide a sensitive early indicator of neural damage in T1DM, before any abnormalities can be identified using standard clinical tests.

## ACKNOWLEDGMENTS

The authors thank the collaborators at the Manchester Diabetes Centre and the Help DiaBEATes campaign in the Salford NHS Foundation Trust and all of the participants in this research.

## Supplementary Material


