# Supplementary material for: Subclinical Auditory Neural Deficits in Patients With Type 1 Diabetes Mellitus
Source: Ear Hear. 2019 Apr 27;41(3):561–75. doi: 10.1097/AUD.0000000000000781 (PMC7664709; doi:10.1097/AUD.0000000000000781)
Supplement: Supplementary file 1 [file aud-41-561-s001.pdf]

Supplemental Digital Content 1: The details of the 30 type 1 diabetes mellitus (T1DM) participants[1].

No. Participant	Participant Sex	T1DM Duration	Diagnosed with Retinopathy	Diagnosed with Neuropathy	Presence of Some Neuropathy Symptoms Confirmed by Participant in the Absence of Clinically Diagnosed Neuropathy
1	F	11	No	Yes	NA
2	M	21	No	Yes	NA
3	F	25	No	No	Numbness and burning pain
4	M	9	Yes	No	Shooting pain and burning pain
5	F	18	No	No	None
6	F	20	Yes	No	Shooting pain and burning pain
7	M	12	Yes	No	None
8	M	15	No	No	None
9	F	18	Yes	No	Numbness and burning pain
10	F	4	No	No	None
11	F	10	No	Yes	NA
12	F	19	Yes	No	Numbness
13	F	8	No	No	None
14	F	25	Yes	No	Burning pain
15	M	20	Yes	No	None
16	F	15	No	Yes	NA
17	F	14	No	No	None
18	F	9	No	No	Burning pain
19	F	24	No	No	Numbness and shooting pain
20	M	28	No	No	Numbness and shooting pain
21	M	12	Yes	No	None
22	M	6	No	No	None
23	F	10	Yes	No	Numbness and shooting pain
24	F	15	No	Yes	NA
25	F	8	No	No	None
26	F	26	No	No	Burning pain
27	F	24	Yes	No	Numbness and burning pain
28	F	26	No	No	Numbness
29	F	16	No	No	None
30	M	18	No	Yes	NA

[1] Listed by duration of type 1 diabetes mellitus (T1DM Duration) in years, and whether or not each had diagnosed clinical neuropathy or retinopathy (self-reported). For each participant with no diagnosed clinical neuropathy, the table also provides the absence or presence, confirmed by the participant, of typical neuropathy symptoms: numbness, shooting pain, burning pain, or none. Not applicable (NA) for participants with diagnosed clinical neuropathy.
