# Supplementary material for: Subclinical Auditory Neural Deficits in Patients With Type 1 Diabetes Mellitus
Source: Ear Hear. 2019 Apr 27;41(3):561–75. doi: 10.1097/AUD.0000000000000781 (PMC7664709; doi:10.1097/AUD.0000000000000781)
Supplement: Supplementary file 2 [file aud-41-561-s002.pdf]

Supplemental Digital Content 2: The details of the 30 matched pairs[1].

[1] Listed by sex, age, experimental group [control or type 1 diabetes mellitus (T1DM)], audiometric threshold of the test ear at 0.5, 1, 2, and 4 kHz, and average audiometric threshold (0.5- 4 kHz).

Pair No.	Sex	Age	Experimental Group	Audiometric Threshold of the Test Ear 0.5 kHz	1 kHz	2 kHz	4 kHz	Average 0.5-4 kHz
1	F	32	Control	0	15	-5	5	3.75
			**T1DM**	5	5	0	5	3.75
2	M	34	Control	0	5	10	10	6.25
			**T1DM**	0	5	5	20	7.5
3	F	27	Control	10	10	5	10	8.75
			**T1DM**	5	10	10	5	7.5
4	M	31	Control	0	10	5	0	3.75
			**T1DM**	5	5	10	5	6.25
5	F	24	Control	5	0	5	5	3.75
			**T1DM**	0	0	0	5	1.25
6	F	22	Control	0	5	5	0	2.5
			**T1DM**	0	5	5	10	5
7	M	24	Control	5	5	5	0	3.75
			**T1DM**	0	0	5	5	2.5
8	M	30	Control	10	15	10	5	10
			**T1DM**	5	5	5	5	5
9	F	21	Control	5	0	10	0	3.75
			**T1DM**	5	5	0	5	3.75
10	F	22	Control	5	5	0	0	2.5
			**T1DM**	5	0	0	0	1.25
11	F	28	Control	15	10	5	0	7.5
			**T1DM**	10	5	0	5	5
12	F	25	Control	0	-5	0	0	-1.25
			**T1DM**	10	5	0	0	3.75
13	F	21	Control	10	5	0	10	6.25
			**T1DM**	5	-5	0	10	2.5
14	F	29	Control	0	-5	0	0	-1.25
			**T1DM**	5	0	5	0	2.5
15	F	30	Control	0	5	5	0	2.5
			**T1DM**	10	5	5	10	7.5
16	F	22	Control	10	5	15	5	8.75
			**T1DM**	5	5	0	5	3.75
17	F	28	Control	5	5	0	0	2.5
			**T1DM**	0	10	10	5	6.25
18	F	20	Control	5	5	5	10	6.25
			**T1DM**	0	5	0	5	2.5
19	F	28	Control	15	0	0	0	3.75
			**T1DM**	10	5	5	5	6.25
20	M	30	Control	0	5	0	5	2.5
			**T1DM**	0	0	0	10	2.5
21	M	19	Control	5	0	-5	-5	-1.25
			**T1DM**	5	5	0	0	2.5
22	M	33	Control	10	10	10	0	7.5
			**T1DM**	20	15	10	5	12.5
23	F	25	Control	10	5	0	10	6.25
			**T1DM**	15	10	10	10	11.25
24	F	18	Control	0	0	0	10	2.5
			**T1DM**	10	5	0	5	5
25	F	21	Control	5	5	0	-5	1.25
			**T1DM**	10	5	5	0	5
26	F	28	Control	10	0	5	5	5
			**T1DM**	15	10	5	10	10
27	F	26	Control	10	10	15	0	8.75
			**T1DM**	20	20	5	10	13.75
28	F	32	Control	0	5	0	0	1.25
			**T1DM**	10	5	0	-5	2.5
29	F	24	Control	10	10	5	0	6.25
			**T1DM**	10	5	5	0	5
30	M	22	Control	0	5	5	10	5
			**T1DM**	5	5	10	15	8.75
