# Supplementary material for: Subclinical Auditory Neural Deficits in Patients With Type 1 Diabetes Mellitus
Source: Ear Hear. 2019 Apr 27;41(3):561–75. doi: 10.1097/AUD.0000000000000781 (PMC7664709; doi:10.1097/AUD.0000000000000781)
Supplement: Supplementary file 3 [file aud-41-561-s003.pdf]

Supplemental Digital Content 3: Statistics for all variables used in the analyses on the auditory brainstem response (ABR) data[1].

ABR measure	Experimental group	Mean	SD	*t/z*	*p*
Wave I amplitude	Control	409.26	389.53	-1.79 (*t*)	0.08
	**T1DM**	318.62	231.82		
Wave III amplitude	Control	459.17	334.27	-1.29 (*t*)	0.21
	**T1DM**	364.92	260.46		
Wave V amplitude	Control	640.67	441.34	-0.75 (*z*)	0.45
	**T1DM**	575.83	349.42		
Wave I-III amplitude ratio	Control	1.24	1.28	0.63 (*z*)	0.53
	**T1DM**	1.41	2.65		
Wave III-V amplitude ratio	Control	0.73	0.59	-0.18 (*z*)	0.86
	**T1DM**	0.79	0.70		
Wave I-V amplitude ratio	Control	0.67	0.60	0.59 (*z*)	0.56
	**T1DM**	0.70	0.43		
Wave I latency	Control	1.69	0.09	1.24 (z)	0.22
	**T1DM**	1.71	0.20		
Wave III latency	Control	3.79	0.22	0.39 (*t*)	0.70
	**T1DM**	3.81	0.23		
Wave V latency	Control	5.45	0.34	1.04 (*t*)	0.31
	**T1DM**	5.53	0.31		
Wave I-III interval	Control	2.10	0.22	0.06 (*t*)	0.95
	**T1DM**	2.10	0.19		
Wave III-V interval	Control	1.66	0.33	0.76 (*t*)	0.45
	**T1DM**	1.72	0.35		
Wave I-V interval	Control	3.76	0.32	0.78 (*t*)	0.44
	**T1DM**	3.81	0.38		

[1] Auditory brainstem response measures (ABR measure): auditory brainstem response peak-to-trough amplitudes (amplitude) [in nV], auditory brainstem response peak-to-trough amplitude ratios (amplitude ratio), auditory brainstem response absolute latencies (latency) [in ms], and auditory brainstem response inter-peak interval (interval) [in ms]. Comparison between the two groups [control or type 1 diabetes mellitus (T1DM)]: standard deviation (SD), and t value from the paired samples *t* test (*t*) or z value from the Wilcoxon signed-ranks test (*z*).
