# Supplementary material for: Subclinical Auditory Neural Deficits in Patients With Type 1 Diabetes Mellitus
Source: Ear Hear. 2019 Apr 27;41(3):561–75. doi: 10.1097/AUD.0000000000000781 (PMC7664709; doi:10.1097/AUD.0000000000000781)
Supplement: Supplementary file 4 [file aud-41-561-s004.pdf]

Supplemental Digital Content 4: Statistics for frequency-following response (FFR) group delay data used in the analyses[1].

**FFR Measure**	**Experimental Group**	**No. Participants**	**Mean**	**SD**	***t***	***p***
FFRadd group delay	Control	17	5.92	3.34	0.66	0.51
	**T1DM**	17	7.03	3.96		
FFRsub lower side band group delay	Control	22	7.30	1.75	0.97	0.35
	**T1DM**	22	7.90	1.94		
FFRsub upper side band group delay	Control	17	7.76	3.96	0.62	0.54
	**T1DM**	17	8.73	5.95		
Mean FFRsub group delay	Control	29	7.46	3.45	1.64	0.11
	**T1DM**	29	8.85	3.67		

[1] Group delay for the addition waveform (FFRadd group delay) [in ms], group delay for the subtraction waveform lower side band (FFRsub lower side band group delay) [in ms], group delay for the subtraction waveform upper side band (FFRsub upper side band group delay) [in ms], and group delay for the mean subtraction waveform (Mean FFRsub group delay) [in ms]. Comparison between the two groups [control or type 1 diabetes mellitus (T1DM)]: number of participants (No. participant), standard deviation (SD), and t value from the paired samples t test (*t*).
