# Supplementary material for: Subclinical Auditory Neural Deficits in Patients With Type 1 Diabetes Mellitus
Source: Ear Hear. 2019 Apr 27;41(3):561–75. doi: 10.1097/AUD.0000000000000781 (PMC7664709; doi:10.1097/AUD.0000000000000781)
Supplement: Supplementary file 5 [file aud-41-561-s005.pdf]

Supplemental Digital Content 5: Correlation between electrophysiological and behavioral measures[1].

---

[1] Asterisks denote a significant difference between the two groups: *$p < 0.05$.

Correlation coefficients (Pearson's (*r*) and Spearman's (*rs*)) and the probability values (*p*) for the correlations between electrophysiological amplitude and latency measures and behavioural measures, for the control and type 1 diabetes mellitus (T1DM) groups. Electrophysiological amplitude measures: auditory brainstem response peak-to-trough amplitudes of wave I (Wave I amplitude) [in nV], auditory brainstem response peak-to-trough amplitudes of wave V (Wave V amplitude) [in nV], signal-to-noise ratios for the frequency-following response addition waveform (FFRadd SNR) [in dB], signal-to-noise ratios for the frequency-following response mean subtraction waveform (Mean FFRsub SNR) [in dB]. Electrophysiological latency measures: auditory brainstem response absolute latency of wave I (Wave I amplitude) [in ms], auditory brainstem response absolute latency of wave V (Wave V amplitude) [in ms], group delay for the frequency-following response addition waveform (FFRadd group delay) [in ms], and group delay for the frequency-following response mean subtraction waveform (Mean FFRsub group delay) [in ms]. Behavioural measures: log- transformed interaural phase difference threshold (IPD threshold) [in log10 Degrees], log- transformed frequency difference limen (FDL) [in log10 percentage], signal-to-noise ratio for 50% correct in the separated speech condition (SNR separated) [in dB], and signal-to-noise ratio for 50% correct in the co-located speech condition (SNR co-located) [in dB].

Measures		Control Group		TIDM Group	
**Electrophysiological Measure**	**Behavioural Measure**	**Correlation *(r/rs)***	***p***	**Correlation *(r/rs)***	***P***
Wave I amplitude	IPD threshold	0.25 (*rs*)	0.19	0.34 (*r*)	0.07
Wave I amplitude	FDL	0.11 (*rs*)	0.55	0.17 (*r*)	0.37
Wave I amplitude	SNR separated	0.20 (*rs*)	0.29	-0.66 (*r*)	0.73
Wave I amplitude	SNR co-located	-0.20 (*rs*)	0.92	0.02 (*r*)	0.90
Wave V amplitude	IPD threshold	0.25 (*rs*)	0.18	-0.17 (*r*)	0.38
Wave V amplitude	FDL	0.19 (*rs*)	0.31	0.01 (*r*)	0.97
Wave V amplitude	SNR separated	-0.20 (*rs*)	0.29	-0.66 (*r*)	0.73
Wave V amplitude	SNR co-located	-0.20 (*rs*)	0.92	-0.23 (*r*)	0.90
FFRadd SNR	IPD threshold	-0.23 (*rs*)	0.22	-0.39 (*r*)	0.03*
FFRadd SNR	FDL	-0.19 (*rs*)	0.32	-0.04 (*r*)	0.83
FFRadd SNR	SNR separated	-0.06 (*rs*)	0.75	0.10 (*r*)	0.60
FFRadd SNR	SNR co-located	-0.17 (*rs*)	0.38	-0.04 (*r*)	0.82
Mean FFRsub SNR	IPD threshold	0.18 (*r*)	0.35	-0.20 (*r*)	0.29
Mean FFRsub SNR	FDL	-0.09 (*r*)	0.65	-0.02 (*r*)	0.90
Mean FFRsub SNR	SNR separated	0.01 (*r*)	0.97	0.14 (*r*)	0.45
Mean FFRsub SNR	SNR co-located	-0.08 (*r*)	0.67	0.02 (*r*)	0.90
Wave I latency	IPD threshold	-0.20 (*r*)	0.29	-0.41 (*rs*)	0.02*
Wave I latency	FDL	0.01 (*r*)	0.98	0.58 (*rs*)	0.001**
Wave I latency	SNR separated	-0.04 (*r*)	0.85	0.12 (*rs*)	0.54
Wave I latency	SNR co-located	-0.10 (*r*)	0.30	0.11 (*rs*)	0.56
Wave V latency	IPD threshold	-0.18 (*r*)	0.35	0.14 (*r*)	0.47
Wave V latency	FDL	-0.20 (*r*)	0.30	-0.04 (*r*)	0.83
Wave V latency	SNR separated	-0.22 (*r*)	0.24	-0.04 (*r*)	0.82
Wave V latency	SNR co-located	-0.33 (*r*)	0.08	-0.09 (*r*)	0.66

FFRadd group delay	IPD threshold	-0.33 (*rs*) (n=27)	0.09	0.07 (*rs*) (n=18)	0.80
FFRadd group delay	FDL	-0.32 (*rs*) (n=27)	0.11	0.04 (*rs*) (n=18)	0.89
FFRadd group delay	SNR separated	-0.16 (*rs*) (n=27)	0.43	-0.14 (*rs*) (n=18)	0.58
FFRadd group delay	SNR co-located	0.13 (*rs*) (n=27)	0.51	-0.20 (*rs*) (n=18)	0.43
Mean FFRsub group delay	IPD threshold	-0.22 (*rs*) (n=30)	0.25	0.26 (*rs*) (n=29)	0.19
Mean FFRsub group delay	FDL	-0.14 (*rs*)	0.46	0.20 (*rs*)	0.30
Mean FFRsub group delay	SNR separated	0.26 (*rs*)	0.10	0.42 (*rs*)	0.02*
Mean FFRsub group delay	SNR co-located	0.00 (*rs*)	0.50	0.28 (*rs*)	0.15
